# Moving Rubber Blade (MRB) for Fouling Control in Anaerobic Ceramic Membrane Bioreactors (AnCMBRs) Treating High-Strength Food Wastewater: Development and Long-Term Application

**DOI:** 10.3390/membranes15060165

**Published:** 2025-06-01

**Authors:** Young-Jae Lee, Hyung-Soo Kim, Hyunsup Jang, Sung-Gwan Park, Ji-Yeon Kim, Sung-Jae Lee, Youngjin Kim, Moon-Hyun Hwang, Sangyoup Lee

**Affiliations:** 1 Department of Water Resources, Graduate School of Water Resources, Sungkyunkwan University, 2066, Seobu-ro, Jangan-gu, Suwon-si 16419, Republic of Korea; lhunjae@skku.edu (Y.-J.L.); sookim@skku.edu (H.-S.K.); 2 Environmental Forensic Laboratory, Sungkyunkwan University, 2066, Seobu-ro, Jangan-gu, Suwon-si 16419, Republic of Korea; janghs@skku.edu; 3 Institute of Conversions Science, Korea University, 145, Anam-ro, Sungbuk-gu, Seoul 02841, Republic of Korea; sunggwan2@korea.ac.kr (S.-G.P.); greentree84@korea.ac.kr (J.-Y.K.); 4 Department of Environmental Engineering, College of Science and Technology, Korea University, 2511, Sejong-ro, Sejong-si 30019, Republic of Korea; samual792@korea.ac.kr (S.-J.L.); kyuksh@korea.ac.kr (Y.K.)

**Keywords:** high-strength food wastewater, membrane fouling control, anaerobic ceramic blade MBR (AnCBMBR), moving rubber blade (MRB), salt-assisted backwash (SAB)

## Abstract

This study investigates membrane fouling control in a submerged anaerobic ceramic membrane bioreactor (AnCMBR) treating high-strength food wastewater (chemical oxygen demand (COD): 10–30 g/L). A hybrid strategy combining mechanical cleaning via a moving rubber blade (MRB) (termed anaerobic ceramic blade MBR (AnCBMBR)) with intermittent salt-assisted backwash (SAB) was tested to manage transmembrane pressure (TMP) and sustain treatment performance. During more than 300 days of field operation, MRB alone maintained stable TMP below 0.15 kg_f_/cm^2^ without backwashing, achieving more than 90% COD removal at a very short hydraulic retention time (HRT) of 1–2 days. Introducing intermittent SAB further stabilized operations and enhanced total phosphorus (T-P) removal by facilitating struvite formation through the interaction of MgCl_2_ and phosphorus in the reactor. The AnCBMBR system demonstrated reliable, long-term fouling control and treatment efficiency, even under high organic loads, proving its viability for small-scale facilities managing concentrated food wastewater. This study advances practical strategies for sustainable anaerobic MBR operation under challenging industrial conditions.

## 1. Introduction

The effective treatment of high-strength organic wastewater, such as food wastewater, presents significant challenges due to its high organic and nutrient content. Since the prohibition of marine disposal of food waste in 2013, the need for efficient and sustainable food wastewater treatment processes has been increasingly emphasized [1]. Anaerobic processes are well known to be suitable for the treatment of such high-strength food wastewater [2,3]. In particular, anaerobic membrane bioreactor (AnMBR) processes offer compact applications by allowing mixed-liquor-(volatile)-suspended-solid (ML(V)SS) concentrations to reach tens of thousands of mg/L, enabling effective treatment of high-strength food wastewater [3]. However, the major limitation of treating high-strength food wastewater using AnMBRs is membrane fouling caused by high organic loading rate (OLR) and ML(V)SS concentrations in the reactor, which results in reduced flux, decreased membrane efficiency, shortened membrane lifespan, and increased operational and maintenance costs [4,5]. Various approaches have been investigated to control membrane fouling in AnMBRs, including chemical and physical membrane cleaning, membrane surface vibration, and media fluidization [6,7,8].

Submerged anaerobic ceramic membrane bioreactors (AnCMBRs) have shown promise in maintaining stable performance under high-strength wastewater conditions due to the robustness and chemical resistance of ceramic membranes [9,10,11,12]. However, membrane fouling still remains a critical issue that negatively impacts system efficiency and longevity. Rotational filtration systems have emerged as a promising alternative to conventional crossflow filtration, offering economic advantages due to improved permeability, which reduces the required membrane module area [13,14]. In addition to the inherent antifouling effect induced by rotational disks, the rotation increases shear forces between suspended media and the membrane surface [15,16]. This effect enhances fouling control compared to conventional gas sparging and pump-driven recirculation techniques. However, most studies have focused on short-term laboratory-scale evaluations, and there is a lack of long-term, real-world data on effective fouling control in AnCMBRs.

To address these persistent challenges, this study introduces a novel moving rubber blade (MRB) mechanism, designed to reciprocate between flat-sheet ceramic membranes in a submerged AnCMBR system. The MRB physically scrapes foulants from the membrane surface, aiming to suppress transmembrane pressure (TMP) buildup and maintain stable filtration performance over extended periods. In addition, the study investigates the synergistic effect of intermittent salt-assisted backwashing (SAB), which combines the benefits of physical cleaning with chemical enhancement through the addition of NaOCl, NaHCO_3_, and MgCl_2_. SAB not only facilitates the removal of fouling layers via osmotic swelling and biofilm disruption but also promotes phosphorus removal by inducing struvite formation, addressing another key challenge in anaerobic treatment processes. What distinguishes this work from previous studies is its long-term, real-scale evaluation of an integrated fouling control approach that combines MRB and SAB, rather than relying solely on conventional chemical or physical cleaning methods. While prior research has explored various fouling control strategies, most have been limited to short-term or laboratory-scale experiments, with few studies demonstrating sustained operation under actual industrial conditions. Here, the effectiveness of the integrated AnCBMBR system was evaluated through over 300 days of continuous operation at a facility treating real food wastewater, with a focus on TMP control, effluent quality, methane production, and microbial community stability. This extended field validation provides practical insights into sustainable fouling control strategies for anaerobic MBRs operating under challenging industrial conditions, demonstrating the potential for application in small-scale facilities and decentralized wastewater management. In summary, this work advances the practical implementation of membrane bioreactor technology for treating high-strength food wastewater by demonstrating robust, long-term fouling control and stable treatment performance, even under high organic loading and short hydraulic retention times. The results contribute to the development of resilient and cost-effective solutions for sustainable food wastewater management, supporting broader efforts to mitigate environmental impacts and promote resource recovery in the wastewater sector.

## 2. Materials and Methods

### 2.1. Configuration of AnCBMBR System

**Figure 1:**
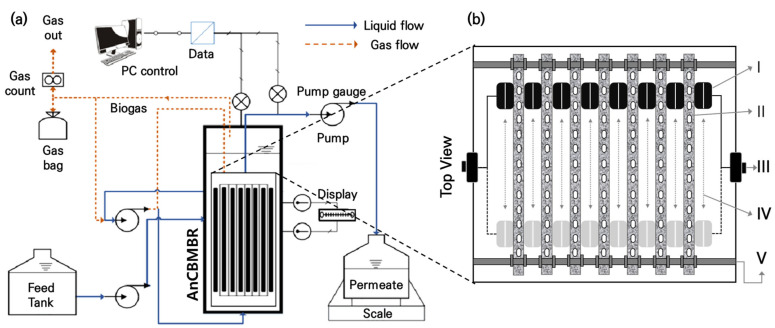
Schematic diagram of the AnCBMBR: (**a**) configuration of AnCBMBR system and (**b**) design of moving rubber blade (MRB) (I. rubber blade, II. Flat-sheet ceramic membrane, III. blade motion device, IV. horizontal reciprocating motion, and V. membrane fixation support).

A submerged AnCMBR system equipped with an MRB (termed AnCBMBR) was designed and implemented to minimize membrane fouling and enhance filtration efficiency. The system incorporated a moving rubber blade (MRB) as a physical fouling control method, which continuously scraped foulants from the membrane surface (See Figure 1a). This MRB method reduced the accumulation of fouling substances and minimized the need for frequent backwashing and chemical cleaning (Figure 1b).

The reactor used in this study had a total volume of 100 L, with a pH 6.6–7.7, a temperature 35 ± 1 °C, and an oxidation–reduction potential (ORP) −400 to −430 mV. The reactor temperature was maintained at 35 ± 1 °C using an automatic temperature control blanket. pH and ORP were continuously monitored with multi-sensor probes (Multi 3630 IDS, Xylem Analytics, Weilheim, Germany), but were not automatically regulated and were allowed to vary according to the anaerobic reactor operation. Reactor mixing was achieved via continuous biogas recirculation, ensuring homogeneous conditions throughout the bioreactor. During long-term experiments (see Section 3.3.1 (Phase IV)), SAB was applied intermittently three times daily (every 8 h) for 5 min per cycle. This newly developed system was designated as an anaerobic ceramic blade membrane bioreactor (AnCBMBR).

For membrane filtration, α-alumina-based ceramic flat-sheet membranes (CH250-1000E02K-5NA, Meiden Singapore Pte. Ltd., Jalan Pesawat, Singapore) were utilized. The specifications of the flat-sheet ceramic membrane used in AnCBMBR system are listed in Table 1. One flat-sheet ceramic membrane allows filtration on both sides, resulting in a filtration area of 670 cm^2^ per membrane. A total of 20 flat-sheet ceramic membranes (total filtration area = 1.34 m^2^) are submerged in the reactor with a volume of 100 L, creating a packing density of 13.4 m^2^/m^3^ (i.e., packing density (m^2^/m^3^) = total filtration area (m^2^)/reactor volume (m^3^)).

### 2.2. Principles and Application of Salt-Assisted Backwashing (SAB)

In this study, the salt-assisted backwashing (SAB) was utilized by combining the backwashing solution with 50 mM NaOCl, 50 mM NaHCO3, and 25 mM MgCl2. These three salts used in SAB penetrate the fouling layer of the membrane in the reactor through backwashing, inducing osmotic swelling [17]. This mechanism weakens the cohesion of the fouling layer, facilitating its removal from the membrane surface.

The reason for selecting these three specific salts is that each salt has its unique role in addition to inducing osmotic swelling. First, NaOCl, which enters the reactor in a diluted state, plays a role in breaking down any biofouling layer that might form on the membrane surface. It should be noted that the SAB solution is significantly diluted upon entering the reactor, and the actual NaOCl concentration is not expected to adversely affect anaerobic microbial activity. This can be corroborated by the stable COD removal and methane production observed during long-term operation (see Section COD Removal Efficiency and Bio Gas Production). MgCl_2_ reacts with the phosphorus in food wastewater to form struvite (i.e., phosphate mineral), which can aid in the removal of phosphorus, which is difficult to remove in anaerobic microbial reactions [18]. Lastly, NaHCO_3_ helps maintain optimal pH conditions in the anaerobic MBR (AnMBR) [19,20]. Therefore, intermittent application of SAB in addition to the main membrane fouling control technique in MBR can help maintain a more stable TMP. Furthermore, although these salts were not expected to affect microbial activity due to their low concentrations (as diluted upon entering the reactor), COD removal efficiency, methane production rate, and changes in the microbial community were systematically analyzed to assess whether SAB impacts microbial activity in the anaerobic reactor.

### 2.3. Operational Conditions of AnCBMBR System

The leachate and condensate used in this study were sourced from the Dongducheon Food Waste Treatment Plant (Dongducheon-si, Gyeonggi-do, Republic of Korea). This facility generates approximately 75 tons/day of food waste leachate and 55 tons/day of condensate during food waste processing. For the operation of the AnCBMBR system, the influent was specifically drawn from the condensate line at this treatment plant, and a dedicated influent line was installed to supply this stream to the AnCBMBR system. The leachate showed COD levels ranging from 150 to 200 g/L, total nitrogen (T-N) from 3.0 to 5.0 g/L, and total phosphorus (T-P) from 0.4 to 0.6 g/L. The condensate generated during the leachate condensation process exhibited COD levels around 10,000 to 50,000 mg/L, T-N levels from 40 to 60 mg/L, and T-P levels from 10 to 30 mg/L. For the initial operation of the AnCBMBR system, considering system stability, a hydraulic retention time (HRT) of 2 days, a COD of 10,000 mg/L, and an MLSS of 20,000 mg/L (MLVSS = 13,000 mg/L) were applied, resulting in an initial food-to-microorganism (F/M) ratio of 0.5 kg-COD/kg-MLSS/day.

After stabilization (approximately 2 months), the system was operated with a COD range increased to 15,000–30,000 mg/L. As the operation continued, the MLSS and MLVSS concentrations gradually increased over time, reaching 28,000 and 22,000 mg/L, respectively, and long-term operations were conducted until the F/M ratio reached 1.07 kg-COD/kg-MLSS/day. Regarding HRT, 2 days were applied during the first 2 months, 1.5 days for the subsequent 2 months, and 1.0 day thereafter.

### 2.4. Analytical Methods

All analytical procedures were conducted in accordance with the guidelines outlined in the Standard Methods for the Examination of Water and Wastewater published by the American Public Health Association (APHA) [21]. The concentrations of the COD, T-N, and T-P were measured using a COD multi-meter (DR6000, HACH, Loveland, CO, USA). MLSS and MLVSS were analyzed using the glass fiber filtration method. Additionally, key operational parameters such as pressure, temperature, and oxidation–reduction potential (ORP) were continuously monitored using a digital pressure gauge with a data logger (DCX-22, Keller Pressure, Winterthur, Switzerland) and temperature/ORP multi-sensors (Multi 3630 IDS, Xylem Analytics, Weilheim, Germany). Biogas produced by microbial reactions within the anaerobic AnCBMBR was quantified, and all collected samples were analyzed within 12 h of sampling to ensure data accuracy and reliability. The composition of biogas generated was analyzed using a gas chromatograph (Agilent 8890 GC with dual FID and Dual S/S, GENTECH Scientific, Arcade, NY, USA).

## 3. Results and Discussion

### 3.1. Optimal Moving Speed of MRB for Fouling Control in AnCBMBR System

**Figure 2:**
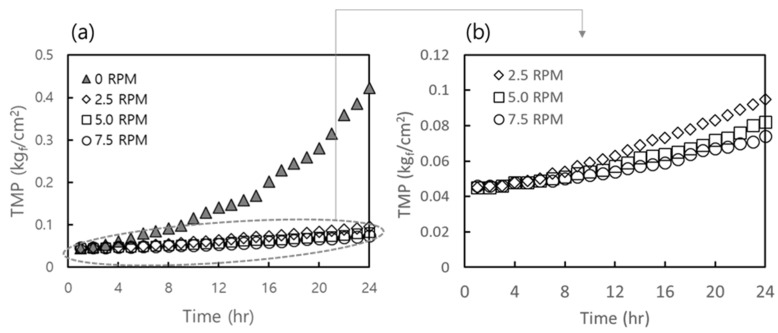
Influence of moving speed (in terms of RPM) of MRB on the suppression of TMP increase during AnCBMBR operation: (**a**) TMP range: 0–0.5 kg_f_/cm² and (**b**) TMP range: 0–0.12 kg_f_/cm².

To determine the optimal moving speed of the moving rubber blade (MRB) for fouling mitigation, the transmembrane pressure (TMP) increase was monitored at different blade moving speeds (i.e., expressed as round per minute (RPM)). Experiments were conducted under the following operating conditions: flux of 6.2 LMH, hydraulic retention time (HRT) of 12 h, temperature of 35 °C, influent COD of 10 g/L, and MLSS and MLVSS concentrations of 20 g/L and 13.9 g/L, respectively. The blade rotation speeds were set at 0, 2.5, 5, and 7.5 RPM, and the corresponding changes in TMP were recorded. The TMP variations at different blade speeds are illustrated in Figure 2.

Without blade operation (0 RPM), TMP increased beyond 0.4 kgf/cm^2^ within 24 h, indicating severe membrane fouling. At 2.5, 5, and 7.5 RPM, the rates of TMP increase were significantly lower. The results showed that at 2.5 RPM, the TMP increase was approximately 6% higher than at 7.5 RPM, while at 5 RPM, the TMP increase was only 1.6% higher than at 7.5 RPM. Although increasing the blade speed resulted in reduced TMP rise, the difference between 5 RPM and 7.5 RPM was minimal (1.6%). Therefore, considering long-term operation and energy efficiency, 5 RPM was selected as the optimal blade speed for fouling control.

### 3.2. Efficiency of Salt-Assisted Backwashing (SAB) on Fouling Mitigation

**Figure 3:**
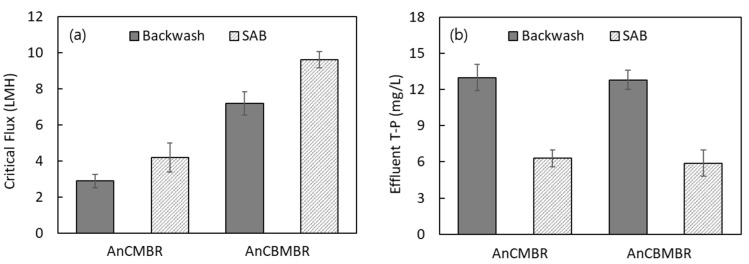
Effect of salt-assisted backwashing (SAB) on (**a**) enhancing critical flux for AnCMBR and AnCBMBR systems and (**b**) lowering T-P concentration in the effluent from AnCMBR and AnCBMBR systems (Note: Error bars indicate the standard deviation from three independent measurements for each data point).

Before long-term operation, the efficiency of SAB was evaluated. The critical flux measurement employing the flux-step method was employed to compare backwashing efficiencies [22,23,24]. All critical flux measurements were conducted in triplicate, and the mean values with standard deviations are presented in Figure 3. The operational cycle was as follows: 15 min of filtration—30 s of pause—1 min of backwashing—30 s of pause—15 min of filtration. Normal backwashing (without salts) and SAB were compared to examine how these different types of backwashing influence the critical flux (Figure 3a). Additionally, phosphorus concentration changes in the effluent were measured to verify the potential for struvite formation during SAB (Figure 3b).

As shown in Figure 3a, when the MRB was not in operation (i.e., AnCMBR), the critical flux was 2.9 LMH with normal backwashing and 4.2 LMH with SAB, indicating that SAB achieved a 45% higher critical flux compared to normal backwashing. When the MRB was operated at 5 RPM (i.e., AnCBMBR), the critical flux increased to 7.2 LMH. Furthermore, the combined application of SAB and MRB operations resulted in a critical flux of 9.6 LMH, demonstrating the synergistic effect of SAB and MRB. These findings suggest that the simultaneous application of SAB and MRB operations effectively enhances the suppression of membrane fouling, making it a promising approach for high HRT operations (i.e., HRT < 24 h).

**Figure 4:**
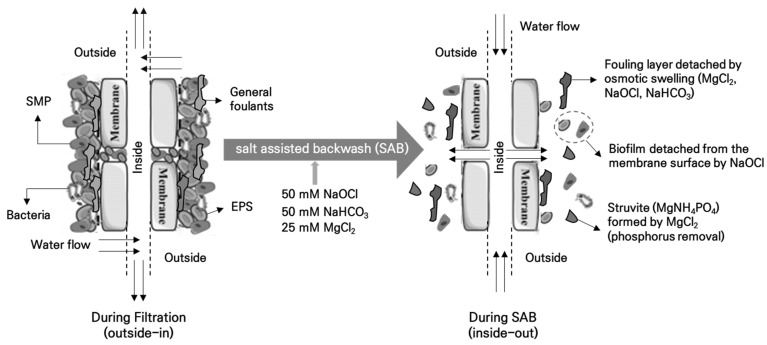
Conceptual mechanisms of salt-assisted backwashing (SAB) and the individual roles of salts in weakening the fouling layer structure on the membrane surface/pores.

As shown in Figure 3b, regardless of MRB application (i.e., both AnCMBR and AnCBMBR), the effluent T-P concentration decreased when SAB was implemented. In both cases, the effluent T-P concentration was approximately 13.0 mg/L with normal backwashing, while it decreased by about 6.0 mg/L when SAB was applied, resulting in a T-P concentration of approximately 6 mg/L. This suggests that the MgCl_2_ included in the SAB solution permeates through the membrane and enters the reactor, where it reacts with phosphorus in the reactor to form struvite [18], leading to a certain degree of decrease in effluent T-P concentration (Figure 3b). The SAB mechanisms proposed in this study are illustrated in Figure 4. However, since the actual formation of struvite was not measured, a confirmation of struvite generation is necessary, and more definitive verification can be achieved when the influent T-P concentration is higher.

### 3.3. Long-Term Operation of AnCBMBR: Fouling Control and System Performance Stability

#### 3.3.1. Fouling Control by MRB and SAB

**Figure 5:**
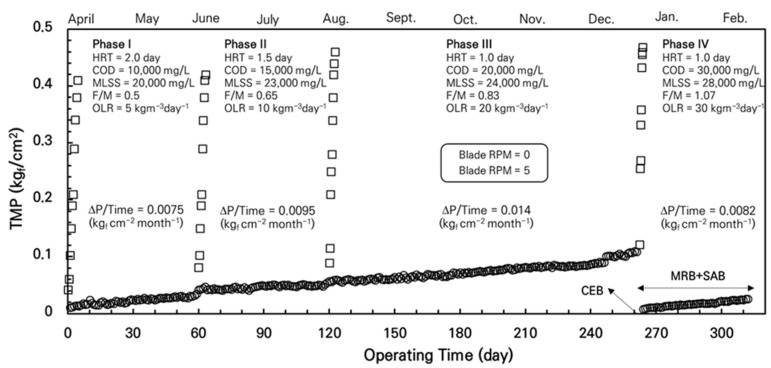
TMP variation during 300 days of AnCBMBR operation under four phases with increasing organic loading and decreasing HRT. Squares (□) represent TMP without blade rotation (RPM = 0), while circles (○) indicate TMP with blade rotation at 5 RPM. Chemical enhanced backwashing (CEB) was performed at day ~260, followed by combined MRB and SAB operation. Fouling rates (i.e., TMP increase rate = ΔP/Time) for each phase are indicated. Operational parameters for each phase are summarized in the inset.

The AnCBMBR system was operated continuously for 300 days under mesophilic conditions (35 ± 1 °C) to evaluate its performance across four sequential operational phases as shown in Figure 5 (Phase I–IV). System parameters, including HRT, COD, MLSS, OLR, and F/M ratio, were systematically modified to assess AnCBMBR process stability (i.e., COD removal) and TMP increase rate (ΔTMP/Time). A constant blade rotation speed of 5 RPM was maintained throughout the study. Backwashing was not conducted in all phases, and only MRB was used to suppress the TMP increase (i.e., membrane fouling control). At the end of Phase III (TMP = 0.15 kgf/cm^2^, approximately 14.7 kPa), chemical enhanced backwashing (CEB) was performed on the membrane. CEB is a maintenance cleaning method involving backwashing combined with chemical agents like acid, caustic, or chlorine to dissolve foulants on membrane surfaces or inside pores. CEB was applied only once during the entire 320-day operational period (i.e., at the end of Phase III), as the primary aim was to evaluate the long-term fouling control efficiency and mechanism of the newly developed MRB and SAB strategies, rather than to compare the cleaning efficacy of individual chemical agents. The comparative effectiveness of various chemical cleaning agents is already well established in the studies [6,7] and was not the main focus of this research. Subsequently, in Phase IV, MBR and SAB were implemented simultaneously to monitor the fouling inhibition effect of the AnCBMBR system when SAB was added to MBR. During this period, SAB was conducted 3 times daily (i.e., every 8 h for 5 min each).

Figure 5 depicts the temporal variation in TMP over the 300-day operation period. At the start of each phase, TMP data measured without MRB (Blade RPM = 0, represented by squares □) exhibited sharp and rapid increases, particularly under shortened HRT conditions. This pronounced TMP escalation in the absence of MRB highlights the rapid progression of membrane fouling under high-strength wastewater conditions when MRB is not applied. Once MRB was activated (Blade RPM = 5, circles ○), TMP increases were substantially mitigated, demonstrating the critical role of MRB in controlling membrane fouling.

Phase I (Days 1–60: Spring season) initiated baseline operation with an HRT of 48 h (2 days), influent COD of 10,000 mg/L, MLSS concentration of 20,000 mg/L, and OLR of 5 kg-COD/m^3^/day. These conditions yielded an F/M ratio of 0.5 kg-COD/kg-MLSS/day. In this phase, the TMP increase rate with MRB was very low at 0.0075 kgf·cm^−2^·month^−1^. This indicates that MRB effectively suppressed membrane fouling, maintaining TMP at minimal levels despite the influent strength. The initial TMP spike observed without MRB further confirms the necessity of mechanical fouling control even under moderate loading.

Phase II (Days 61–120) intensified operational parameters by reducing HRT to 36 h (1.5 days) while increasing influent COD to 15,000 mg/L and MLSS to 23,000 mg/L. This adjustment elevated the OLR to 10 kg-COD/m^3^/day and F/M ratio to 0.65 kg-COD/kg-MLSS/day. In this phase, the TMP increase rate rose slightly to 0.0095 kgf·cm^−2^·month^−1^. Despite these more challenging conditions, MRB continued to effectively control fouling, preventing excessive TMP buildup. The clear contrast between the rapid TMP rise without MRB and the controlled TMP with MRB underscores its robustness in fouling mitigation under elevated OLR and F/M.

Phase III (Days 121–270) further optimized loading capacity through HRT reduction to 24 h (1 day), with concurrent increases in influent COD (20,000 mg/L), MLSS (24,000 mg/L), and OLR (20 kg-COD/m^3^/day), achieving an F/M ratio of 0.83 kg-COD/kg-MLSS/day. In this phase, MRB was still able to delay fouling progression for a significant duration. However, during the latter 20 days of this 150-day phase, TMP progressively exceeded the criteria established in this study of 0.10 kgf/cm^2^ (see Figure 2), prompting a CEB prior to Phase IV. The procedure of CEB was taken from the common methods described in the previous studies [25,26].

In Phase IV (Days 271–310), following CEB at the end of Phase III, the system was subjected to maximum loading (HRT = 1.0-day, COD = 30,000 mg/L, MLSS = 28,000 mg/L, OLR = 30 kg-COD/m^3^/day, F/M = 1.07). Under these extreme conditions, the combined application of MRB and SAB (three 5 min cycles daily) markedly suppressed TMP increase, reducing the fouling rate to 0.0082 kgf·cm^−2^·month^−1^. This fouling rate is comparable to that observed in Phase I, despite the six-fold increase in OLR and doubling of the F/M ratio, clearly demonstrating the synergistic effectiveness of SAB and MRB in maintaining membrane permeability and controlling fouling under highly challenging operational conditions.

The sharp TMP increases observed at the start of each phase without MRB provide direct evidence that membrane fouling progresses rapidly under short HRT and high-strength wastewater conditions if MRB is not applied. This rapid fouling onset emphasizes the vulnerability of the membrane system and the necessity of mechanical fouling control strategies. MRB alone effectively controlled fouling during Phases I to III, even as OLR and F/M increased, by physically disrupting foulant accumulation on the membrane surface. However, at the highest loading in Phase III, MRB’s efficacy was limited over prolonged operation, necessitating CEB to chemically remove irreversible foulants. The addition of SAB in Phase IV provided an auxiliary chemical fouling control that complemented the continuous mechanical action of MRB, preventing compaction and buildup of foulants more effectively than MRB alone. This combined approach stabilized TMP at low levels, enabling operation at the highest organic loads tested without rapid fouling.

The fouling rate exhibited a strong positive correlation with OLR and F/M ratio, indicating that increased substrate availability per biomass unit enhances the production of soluble microbial products (SMPs) and extracellular polymeric substances (EPSs), which exacerbate membrane fouling [27,28,29]. The relatively modest increase in MLSS compared to OLR suggests that biomass activity and metabolic byproduct generation, rather than biomass concentration alone, are primary drivers of fouling. In addition, despite spanning multiple seasons, the controlled mesophilic temperature (35 ± 1 °C) ensured minimal influence of ambient temperature fluctuations on microbial activity and fouling behavior, supporting the robustness of the AnCBMBR system under stable thermal conditions.

#### 3.3.2. Influence of MBR and SAB on the Performance of AnCBMBR

##### MLSS and MLVSS

The observed increase in MLSS and MLVSS concentrations over the operational period (310 days) indicates a stable microbial community and biomass accumulation in the reactor [2,3]. Initially, MLSS was seeded at 20 g/L and gradually increased to 28 g/L by the end of the operation, while MLVSS increased from 13 g/L to 20 g/L. The MLVSS/MLSS ratio increased from 65% (Phase I) to 71% (Phase IV), suggesting an improvement in biomass activity and organic degradation potential [30,31].

**Figure 6:**
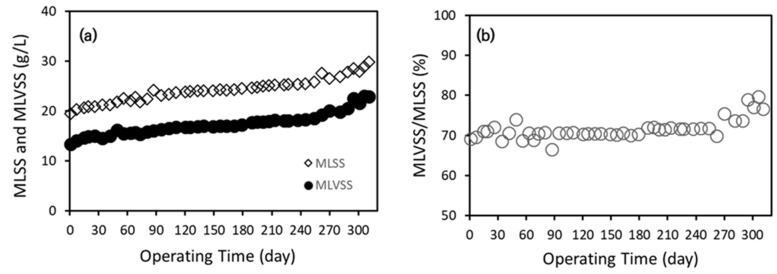
Long-term performance of AnCBMBR system in terms of (**a**) MLSS and MLVSS concentrations and (**b**) ratio of MLVSS to MLSS.

This increase in MLVSS proportion demonstrates that biodegradable organic matter was effectively converted into microbial biomass rather than non-degradable suspended solids accumulation. Typically, in anaerobic bioreactors, an MLVSS/MLSS ratio above 65% is considered indicative of efficient microbial activity and long-term process stability [32,33]. The gradual increase in this ratio throughout the operation suggests favorable conditions for anaerobic microbial growth and organic matter degradation efficiency. These trends are illustrated in Figure 6.

##### COD Removal Efficiency and Bio Gas Production

**Figure 7:**
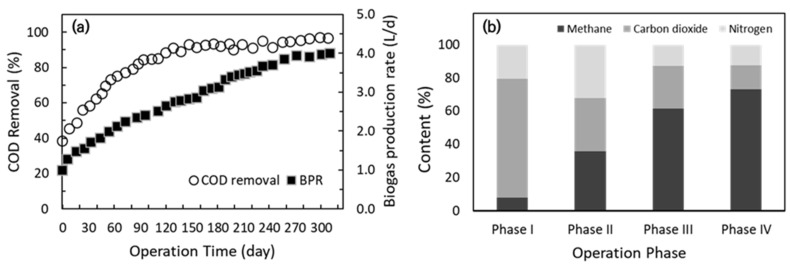
Long-term performance of AnCBMBR system in terms of (**a**) COD removal efficiency and biogas production rate (BPR) and (**b**) biogas composition with respect to the operation phases.

The long-term operation of the AnCBMBR system demonstrated a continuous improvement in COD removal efficiency and biogas production as shown in Figure 7. Initially, COD removal was approximately 40%, but increased steadily to 75% after 2 months and surpassed 90% after 5 months. Following this acclimation period, COD removal efficiency remained consistently above 90% for the remainder of the 300-day operation. This indicates that the system achieved stable and high-level biodegradation of organic matter in high-strength food wastewater even with a very short HRT of 1–2 days. Importantly, the application of the mechanical fouling control strategy (i.e., MRB) did not adversely affect the anaerobic COD removal process, confirming that physical agitation can be integrated without compromising biological treatment performance.

Biogas production rate (BPR) exhibited a parallel upward trend (Figure 7a). Biogas output was first observed at 1.0 L/d after 12 days, increasing to 2.0 L/d by Day 30 and 2.5 L/d after 3 months. As the system stabilized and organic loading increased, biogas production further rose to 3.0 L/d after 5 months and ultimately reached approximately 4.0 L/d by the end of the operation. This progressive increase is consistent with the enhanced COD removal and reflects the system’s improved metabolic activity over time.

Analysis of biogas composition (Figure 7b) revealed a marked shift in methane content as the operation progressed. During the initial phase, methane accounted for only 7.6% of the biogas, with high proportions of CO_2_ (71.7%) and N_2_ (20.7%). As the microbial community adapted and the anaerobic digestion process matured, methane content increased to 35% by Day 90, 61% by Day 150, and reached 73% by Day 300. This trend indicates a successful enrichment of methanogenic populations and a transition toward more efficient methane conversion, resulting in improved biogas quality suitable for energy recovery.

These results collectively demonstrate that the AnCBMBR system, even under intensified operational conditions and with continuous MRB application, can achieve robust and stable COD removal alongside efficient biogas production. The progressive increase in methane fraction further highlights the system’s capability for effective anaerobic digestion and energy recovery. Notably, the integration of MRB for membrane fouling control did not impair the core biological processes, supporting its practical applicability for long-term, high-strength wastewater treatment. In addition, the simultaneous improvement in both COD removal and methane yield suggests that the system maintained a balanced microbial ecosystem, with sufficient syntrophic interactions between fermentative bacteria and methanogens. This balance is critical for stable reactor performance, especially under high organic loading, and underscores the resilience of the AnCBMBR configuration to operational intensification.

##### T-N and T-P

**Figure 8:**
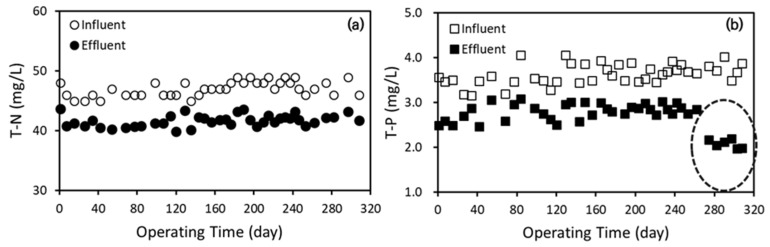
Long-term performance of AnCBMBR system in terms of (**a**) total nitrogen (T-N) and (**b**) total phosphorus (T-P) (in (**b**), the reduced effluent T-P concentration after SAB application is indicated by the dotted circle).

The nutrient concentrations of total nitrogen (T-N) and total phosphorus (T-P) in the influent and effluent of the AnCBMBR system over approximately 300 days of operation are presented in Figure 8. The influent T-N concentration remained relatively stable, averaging around 47–50 mg/L, while the effluent T-N concentration fluctuated narrowly between 38 and 43 mg/L throughout the operational period (Figure 8a). Similarly, influent T-P concentrations ranged between approximately 3.5 and 4.2 mg/L, while effluent T-P concentrations were generally lower, fluctuating between 2.0 and 3.0 mg/L during most of the operation (Figure 8b). These modest reductions of approximately 10–15% and 20–35% in T-N and T-P, respectively, indicate limited nutrient removal by the AnCBMBR process. This observation aligns with the fundamental characteristics of anaerobic biological processes, which are primarily designed for the anaerobic degradation of high-strength organic matter rather than nutrient removal. Effective nitrogen and phosphorus removal typically require additional biological or chemical treatment steps, such as aerobic-anoxic-oxic (A2O) processes, chemical precipitation, or other advanced nutrient removal technologies [34,35,36].

The slight decrease in T-N and T-P concentrations in the effluent compared to the influent can be attributed to membrane-specific effects inherent to membrane bioreactor (MBR) technology. Unlike conventional biological treatment, MBR systems employ physical separation mechanisms, where interactions such as steric hindrance and active membrane sieving may partially retain particulate or colloidal nutrient forms, resulting in modest nutrient reductions. This partial retention effect, however, is generally insufficient to meet stringent nutrient discharge limits, reinforcing the need for complementary nutrient removal processes.

A notable change in T-P concentration was observed following the chemical enhanced backwashing (CEB) event at approximately Day 270, after which salt-assisted backwashing (SAB) was introduced alongside the moving rubber blade (MRB) to better control membrane fouling. Post-CEB and SAB implementation, effluent T-P concentrations showed a significant decline compared to earlier operation periods (Figure 8b). This decrease likely reflects the formation and precipitation of struvite (magnesium ammonium phosphate), facilitated by the presence of MgCl_2_ in the SAB solution and its interaction with phosphorus in the reactor. The struvite precipitation not only contributes to phosphorus removal but may also aid in mitigating membrane fouling by reducing soluble phosphorus concentrations.

While this finding suggests a promising avenue for phosphorus removal linked to SAB operation, it is important to note that this study primarily focused on demonstrating SAB’s effectiveness in membrane fouling control. Therefore, further targeted investigations are warranted to elucidate the mechanisms, optimize the conditions, and quantify the T-P removal potential associated with struvite formation in AnCBMBR systems employing SAB.

### 3.4. Operational Insights of the AnCBMBR

Long-term operation of the AnCBMBR system under real food wastewater conditions provided valuable insights into process stability, fouling management, and nutrient dynamics. The AnCMBR equipped with the moving rubber blade (MRB) (i.e., AnCBMBR) proved highly effective for physical fouling control, maintaining TMP below 0.15 kgf/cm^2^ for extended periods even as organic loading rates (OLRs) increased from 5 to 30 kg-COD/m^3^/day and with a relatively very short HRT (i.e., 1.0 to 2.0 days). This allowed for stable operation at high mixed-liquor-suspended-solid (MLSS) concentrations (up to 28,000 mg/L) and food-to-microorganism (F/M) ratios exceeding 1.0, conditions that are particularly challenging and commonly encountered in small-scale, high-strength food wastewater treatment facilities where anaerobic processes must be effectively applied to manage concentrated organic loads. Importantly, the application of MRB and SAB in this study did not adversely impact COD removal or biogas production, confirming their compatibility with anaerobic biological processes. This is a significant advantage, as several previous studies have reported that certain physical cleaning methods such as high-intensity gas sparging, ultrasonic cleaning, or abrasive scouring can disrupt microbial metabolism, leading to decreased COD removal efficiency and reduced biogas yields in anaerobic membrane bioreactors [15,37,38].

The integration of intermittent SAB with MRB yielded synergistic benefits: the combined approach not only further suppressed TMP increases and enhanced critical flux (from 7.2 to 9.6 LMH), but also contributed to a marked reduction in effluent T-P concentration. The observed decrease in T-P, particularly after SAB initiation, is attributed to struvite precipitation facilitated by MgCl_2_ addition, as evidenced by effluent T-P dropping approximately 30% during SAB operation. This dual fouling and phosphorus control mechanism is especially advantageous for food wastewater, which often contains high phosphorus loads [3,9,13].

Despite these advances, the AnCBMBR system alone provided only limited removal of T-N and T-P (typically 10–15% and 20–40%, respectively), due to the absence of aerobic or anoxic zones required for biological nutrient removal. The partial retention of nutrients is likely due to membrane sieving effects, but this is insufficient for regulatory compliance [31,36]. Therefore, integration with downstream nutrient removal processes (e.g., aerobic polishing, chemical precipitation, or A2O systems) is recommended for full-scale applications targeting comprehensive nutrient removal.

The AnCBMBR system also demonstrated stable biogas production, with daily yields increasing to 4.0 L/d and methane content rising from 7.6% to 73% over the operational period, confirming robust anaerobic digestion and energy recovery potential. Importantly, MRB and SAB did not negatively affect COD removal or biogas yield, confirming the compatibility of these fouling control strategies with core biological processes.

## 4. Conclusions

This study demonstrated the long-term effectiveness of a submerged anaerobic ceramic membrane bioreactor equipped with a moving rubber blade (AnCBMBR) and intermittent salt-assisted backwash (SAB) for treating high-strength food wastewater. Over 300 days of continuous field operation, the system consistently maintained stable TMP, with MRB alone keeping TMP below 0.15 kg_f_/cm^2^ and SAB further enhancing fouling mitigation and membrane performance.

The AnCBMBR achieved over 90% COD removal at short hydraulic retention times (1–2 days), even as ORL increased up to 30 kg-COD/m^3^/day and MLSS reached 28,000 mg/L. Biogas production steadily increased, reaching 4.0 L/d with methane content up to 73%, underscoring the system’s potential for energy recovery from food waste streams.

The integration of SAB with MRB provided clear operational advantages. SAB increased the critical flux by 45% compared to conventional backwashing, and the combined approach achieved a critical flux of 9.6 LMH. Additionally, SAB contributed to a noticeable reduction in effluent T-P concentration, likely due to struvite precipitation induced by MgCl_2_ addition. This dual benefit of fouling and phosphorus control is particularly valuable for food wastewater treatment.

While the system achieved only a modest removal of T-N and T-P due to the absence of biological nutrient removal pathways under anaerobic conditions, it maintained high COD removal and stable biogas production, confirming its compatibility with anaerobic processes. The AnCBMBR exhibited resilience and operational stability under variable and challenging influent conditions, supporting its applicability for small-scale or decentralized treatment of concentrated food wastewater.

Overall, this study provides a practical and scalable strategy for membrane fouling management in AnCBMBR systems. The combined use of MRB and SAB enables robust, long-term operation with high organic removal and energy recovery, while also offering a pathway for enhanced phosphorus control. While these results highlight the viability of the AnCBMBR for pilot-scale applications treating concentrated food wastewater, further studies are required to evaluate scalability, energy efficiency, and operational challenges in full-scale facilities. The findings advance sustainable anaerobic MBR technology by providing a framework for fouling control and resource recovery in small-to-medium-scale installations, though site-specific optimizations would be necessary for broader implementation across diverse operational environments.

## Figures and Tables

**Table 1:** Cases for model comparison based on MBR process data and target parameters.

Membrane Property	Specifications
Material	SiC-based ceramic
Size	Length: 23.1 cm, Width: 14.5 cm
Pore size	0.1 μm
Thickness	0.55 cm
Filtration area (both sides)	670 cm^2^
Pure water permeability	40 m^3^/(m^2^·day) (100 kPa, 25 °C)

## Data Availability

The data presented in this study are available on request from the corresponding authors.

## References

[1] Shrivastava V., Ali I., Marjub M.M., Rene E.R., Soto A.M.F. (2022). Wastewater in the food industry: Treatment technologies and reuse potential. Chemosphere.

[2] Chernicharo C.A.L. (2006). Post-Treatment Options for the Anaerobic Treatment of Domestic Wastewater. Rev. Environ. Sci. Biotechnol..

[3] Pavón-Silva T., Pacheco-Salazar V., Sánchez-Meza J.C., Roa-Morales G., Colín-Cruz A. (2009). Physicochemical and biological combined treatment applied to a food industry wastewater for reuse. J. Environ. Sci. Health A.

[4] Jeong Y., Hermanowicz S.W., Park C. (2017). Treatment of food waste recycling wastewater using anaerobic ceramic membrane bioreactor for biogas production in mainstream treatment process of domestic wastewater. Water Res..

[5] Kim J., Shin J., Kim H., Lee J.-Y., Yoon M.-h., Won S., Lee B.-C., Song K.G. (2014). Membrane fouling control using a rotary disk in a submerged anaerobic membrane sponge bioreactor. Bioresour. Technol..

[6] Erkan H.S., Turan N.B., Engin G.O., Mannina G., Pandey A., Larroche C., Ng H.Y., Ngo H.H. (2020). Fouling control in MBR in a sustainable perspective. Current Developments in Biotechnology and Bioengineering.

[7] Kola A., Ye Y., Le-Clech P., Chen V. (2014). Transverse vibration as novel membrane fouling mitigation strategy in anaerobic membrane bioreactor applications. J. Membr. Sci..

[8] Ngo H.-H., Guo W., Xing W. (2008). Evaluation of a novel sponge-submerged membrane bioreactor (SSMBR) for sustainable water reclamation. Bioresour. Technol..

[9] Ramos C., García A., Diez V. (2014). Performance of an AnMBR pilot plant treating high-strength lipid wastewater: Biological and filtration processes. Water Res..

[10] Jaffrin M.Y. (2012). Dynamic filtration with rotating disks, and rotating and vibrating membranes: An update. Curr. Opin. Chem. Eng..

[11] Ríos S.D., Salvadó J., Farriol X., Torras C. (2012). Antifouling microfiltration strategies to harvest microalgae for biofuel. Bioresour. Technol..

[12] Kim J., Kim K., Ye H., Lee E., Shin C., McCarty P.L., Bae J. (2011). Anaerobic Fluidized Bed Membrane Bioreactor for Wastewater Treatment. Environ. Sci. Technol..

[13] Lee W.-N., Kang I.-J., Lee C.-H. (2006). Factors affecting filtration characteristics in membrane-coupled moving bed biofilm reactor. Water Res..

[14] Jamal Khan S., Zohaib Ur R., Visvanathan C., Jegatheesan V. (2012). Influence of biofilm carriers on membrane fouling propensity in moving biofilm membrane bioreactor. Bioresour. Technol..

[15] Lin H., Peng W., Zhang M., Chen J., Hong H., Zhang Y. (2013). A review on anaerobic membrane bioreactors: Applications, membrane fouling and future perspectives. Desalination.

[16] Jaffrin M.Y., Ding L.-H., Akoum O., Brou A. (2004). A hydrodynamic comparison between rotating disk and vibratory dynamic filtration systems. J. Membr. Sci..

[17] Lee S., Elimelech M. (2007). Salt cleaning of organic-fouled reverse osmosis membranes. Water Res..

[18] Leng Y., Loares A. (2021). Understanding the mechanisms of biological struvite biomineralization. Chemosphere.

[19] Mo W., Soh L., Werber J.R., Elimelech M., Zimmerman J.B. (2015). Application of membrane dewatering for algal biofuel. Algal Res..

[20] Gkotsis P.K., Banti D.C., Peleka E.N., Zouboulis A.I., Samaras P.E. (2014). Fouling Issues in Membrane Bioreactors (MBRs) for Wastewater Treatment: Major Mechanisms, Prevention and Control Strategies. Processes.

[21] (2017). Standard Methods for the Examination of Water and Wastewater.

[22] Le Clech P., Jefferson B., Chang I.S., Judd S.J. (2003). Critical flux determination by the flux-step method in a submerged membrane bioreactor. J. Membr. Sci..

[23] Du X., Shi Y., Jegatheesan V., Haq I.U. (2020). A Review on the Mechanism, Impacts and Control Methods of Membrane Fouling in MBR System. Membranes.

[24] Rahman T.U., Roy H., Islam M.R., Tahmid M., Fariha A., Mazumder A., Tasnim N., Pervez M.N., Cai Y., Naddeo V. (2023). The Advancement in Membrane Bioreactor (MBR) Technology toward Sustainable Industrial Wastewater Management. Membranes.

[25] Meng F., Chae S.R., Drews A., Kraume M., Shin H.S., Yang F. (2009). Recent advances in membrane bioreactors (MBRs): Membrane fouling and membrane material. Water Res..

[26] Ramos C., Zecchino F., Ezquerra D., Diez V. (2014). Chemical cleaning of membranes from an anaerobic membrane bioreactor treating food industry wastewater. J. Membrane Sci..

[27] Iorhemen O.T., Hamza R.A., Tay J.H. (2016). Membrane Bioreactor (MBR) Technology for Wastewater Treatment and Reclamation: Membrane Fouling. Membranes.

[28] Medina S.C., Zamora-Vacca N., Luna H.J., Ratkovich N., Rodríguez Susa M. (2020). SMP Production in an Anaerobic Submerged Membrane Bioreactor (AnMBR) at Different Organic Loading Rates. Membranes.

[29] Mallah N.B., Shah A.A., Pirzada A.M., Ali I., Khan M.I., Jatoi A.S., Ullman J.L., Mahar R.B. (2024). Advanced Control Strategies of Membrane Fouling in Wastewater Treatment: A Review. Processes.

[30] Gkotsis P.K., Zouboulis A.I. (2019). Biomass Characteristics and Their Effect on Membrane Bioreactor Fouling. Molecules.

[31] Luo B., Liu Y., Zhang Q., Yan Y., He H., Wang Y., Yang X., Li J., Huang W., Xu J. (2024). Study on Influencing Factors and Chemical Kinetics in the High-Concentration Simultaneous Nitrification and Denitrification (SND) Process. Water.

[32] Nilusha R.T., Yu D., Zhang J., Wei Y. (2020). Effects of Solids Retention Time on the Anaerobic Membrane Bioreactor with Yttria-Based Ceramic Membrane Treating Domestic Wastewater at Ambient Temperature. Membranes.

[33] Jiang Z., Xia Z., Liu S., Wei Q., Fan H., Qi L., Liu G., Wang H. (2025). The effect of fine grits and fine debris concentrations on the MLVSS/MLSS ratio of an activated sludge system. J. Environ. Sci..

[34] Malila R., Lehtoranta S., Viskari E.-L. (2019). The Role of Source Separation in Nutrient Recovery—Comparison of Alternative Wastewater Treatment Systems. J. Clean. Prod..

[35] Czerwionka K., Wilinska A., Tuszynska A. (2020). The Use of Organic Coagulants in the Primary Precipitation Process at Wastewater Treatment Plants. Water.

[36] Li J., Dong K., Bai S., Fan Y., Feng Y., Liang M., Wang D. (2023). Efficacy of Nitrogen and Phosphorus Removal and Microbial Characterization of Combined A2O-MBBR Constructed Wetlands. Water.

[37] Millanar-Marfa J.M.J., Borea L., De Luna M.D.G., Ballesteros F.C., Belgiorno V., Naddeo V. (2018). Fouling Mitigation and Wastewater Treatment Enhancement through the Application of an Electro Moving Bed Membrane Bioreactor (eMB-MBR). Membranes.

[38] Kaya R., Ersahin M.E., Ozgun H., Kose-Mutlu B., Pasaoglu M.E., Koyuncu I. (2023). Vibratory membrane bioreactor systems in wastewater treatment: A short review. J. Water Process Eng..

